# Predictors of neonatal near-misses in Worabe Comprehensive Specialized Hospital, Southern Ethiopia

**DOI:** 10.3389/fped.2024.1326568

**Published:** 2024-05-31

**Authors:** Shemsu Yasin, Lemesa Abdisa, Hirbo Shore Roba, Abera Kenay Tura

**Affiliations:** ^1^ Sankura Woreda Health Office, Alem Gebeya, Central Ethiopia, Ethiopia; ^2^ School of Nursing and Midwifery, College of Health and Medical Sciences, Haramaya University, Harar, Ethiopia; ^3^ School of Public Health, College of Health and Medical Sciences, Haramaya University, Harar, Ethiopia; ^4^ Department of Obstetrics and Gynecology, University Medical Center Groningen, University of Groningen, Groningen, Netherlands

**Keywords:** neonatal near-miss, predictors, determinant, morbidity, Southern Ethiopia

## Abstract

**Background:**

Neonatal deaths are still a major leading cause of social and economic crises. Identifying neonatal near-miss events and identifying their predictors is crucial to developing comprehensive and pertinent strategies to alleviate neonatal morbidity and death. However, neither neonatal near-miss events nor their predictors were analyzed in the study area. Therefore, this study is aimed at assessing the predictors of neonatal near-misses among neonates born at Worabe Comprehensive Specialized Hospital, Southern Ethiopia, in 2021

**Methods:**

A hospital-based unmatched case-control study was conducted from 10 November 2021 to 30 November 2021. A pre-tested, structured, and standard abstraction checklist was used to collect the data. After checking the data for completeness and consistency, it was coded and entered into Epi-Data 3.1 and then exported to Stata version 14 for analysis. All independent variables with a *p*-value ≤0.25 in bivariable binary logistic regression were entered into a multivariable analysis to control the confounding. Variables with *p*-values <0.05 were considered statistically significant.

**Results:**

In this study, 134 neonatal near-miss cases and 268 controls were involved. The identified predictors of neonatal near-misses were rural residence [adjusted odds ratio (AOR): 2.01; 95% confidence interval (CI): 1.31–5.84], no antenatal care (ANC) follow-up visits (AOR: 2.98; 95% CI: 1.77–5.56), antepartum hemorrhage (AOR: 2.12; 95% CI: 1.18–4.07), premature rupture of the membrane (AOR: 2.55; 95% CI: 1.54–5.67), and non-vertex fetal presentation (AOR: 3.05; 95% CI: 1.93–5.42).

**Conclusion:**

The current study identified rural residents, no ANC visits, antepartum hemorrhage, premature rupture of membrane, and non-vertex fetal presentation as being significantly associated with neonatal near-miss cases. As a result, local health planners and healthcare practitioners must collaborate in enhancing maternal healthcare services, focusing specifically on the early identification of issues and appropriate treatment.

## Introduction

The term “neonatal near-miss” (NNM) refers to a situation in which a newborn is on the verge of dying but survives the neonatal period (1). Neonatal mortality is one of the key indicators of social, economic, and healthcare progress (2). Around one-third and three-quarters of neonatal mortalities occur the first week and the first month after birth, respectively (3, 4). Globally, there were more than 2.7 million under-five mortality cases in 2017, with about 1 million (or 37%) of those deaths occurring in newborns during the first seven days of birth (5).

Neonatal mortality and morbidity continue to be a global public health issue, with the majority of instances occurring in middle- and low-income nations (MLIC) (4, 6); South Central Asia and sub-Saharan Africa accounted for the majority of this figure (7, 8). Neonatal mortality accounted for more than three-fifths of infant mortality and two-fifths of under-five deaths in Ethiopia (9). Per the 2019 Ethiopia Mini Demographic Health Survey, the mortality rate for newborns stands at 30 deaths per 1,000 live births (10).

NNM affects early childhood development and wellness, including behavioral issues, educational capacity, medical conditions, and a higher likelihood of dependency (11). Neonates who are exposed to life-threatening situations may develop long-term complications like neurological, eye, and ear problems as well as chronic diseases like diabetes mellitus, heart diseases, chronic lung disease, and hearing loss in later life (12).

From 2016 to 2030, the Sustainable Development Goals (SDG) of the United Nations aimed to eliminate unnecessary infant deaths, with a target of less than 12 per 1,000 live births by the year 2030 (13). The neonatal mortality rate remains a significant focus for the government because it accounts for the majority of deaths among children under the age of 5 (14). According to the Ethiopian Demographic Health Survey 2019 report, the Ethiopian neonatal mortality rate was 33 per 1,000 live births. With the current neonatal mortality rate trajectory, Ethiopia will not be able to meet the 2030 SDG objective for controlling the neonatal mortality rate (15).

Different studies in Ethiopia and abroad reported predictors of neonatal near-misses like maternal age greater than 35 years, lack of antenatal care (ANC) contact, less than the recommended number of ANC visits, maternal life-threatening conditions, cesarean-section deliveries, maternal near-misses, gestational age prenatal asphyxia, Apgar score less than 7, cesarean section delivery, primiparity, neonatal sepsis, low birth weight, antepartum hemorrhage (APH), maternal syphilis, and maternal chronic diseases like hypertension and diabetes mellitus (16–18).

The emphasis of the Global Maternal Child Survival Program in Ethiopia is on newborns. It works alongside the Ethiopian government to enhance community-based maternal, neonatal, and child health services. The goals include promoting health-seeking behaviors, implementing top-quality newborn care practices like managing newborn sepsis, and strengthening district capacity through comprehensive support systems (19). Identifying neonatal near-miss events and addressing their predictors is essential for devising comprehensive and relevant interventions to tackle neonatal morbidity and mortality. However, neither neonatal near-misses nor their predictors have been adequately investigated, specifically within the study area. Therefore, this study aimed to assess the predictors of neonatal near-misses among neonates born at the Worabe Comprehensive Specialized Hospital, Southern Ethiopia, in 2021, over a period of nearly 3 weeks.

## Materials and methods

### Study setting and design

A hospital-based unmatched case-control study was conducted among neonates admitted at the Worabe Comprehensive Specialized Hospital, located in Worabe Town, Silte Zone, Southern Nations, Nationalities, and Peoples’ Region of Ethiopia. It is located 172 km southwest of Addis Ababa. The hospital provides healthcare services to more than 1.5 million people and is a referral center for three primary hospitals and 34 health centers. It gives emergency, out-patient, and in-patient specialized care in medical, surgical, pediatrics, gynecology, obstetrics, psychiatry, and neonatal intensive care unit (NICU) wings. The neonatal intensive care unit has 18 beds, 5 incubators, 15 radiant warmers, and 23 staff: 14 Bachelor Science (BSc) and 3 diploma nurses, 2 BSc midwifery, 1 general practitioner, 2 consultant senior pediatricians, 1 consultant obstetrician, and 1 gynecologist. The neonatal intensive care unit offers diagnostics and treatment for approximately 1,200 babies per year, from its approximately 3,000 deliveries. The patients’ medical record notes from 28 June 2020 to 27 June 2021 were extracted during the study period from 10 November 2021 to 30 November 2021.

### Study population

#### Cases

The Latin American Center for Perinatology (CLAP)’s definition of a neonatal near-miss was used to select cases. That is, neonates who have one or more of the following characteristics: birth weight less than 1.7 kg, Apgar score less than 7, gestational age less than 33 weeks, and/or parenteral antibiotic treatment, nasal continuous positive airway pressure (CPAP), any intubation during the first week of life, phototherapy within 24 h of birth, cardiopulmonary resuscitation (CPR), vasoactive drug use, anticonvulsants use, surfactant use, blood product use, steroid use, and surgery (20).

#### Control

Neonates hospitalized for non-near-miss neonatal illnesses, as well as neonates admitted for post-natal care treatment, were included as the control group.

### Eligibility criteria

#### Inclusion criteria

All live birth neonates who delivered in Worabe Comprehensive Specialized Hospital from notes from 28 June 2020 to 27 June 2021, were included in the study.

#### Exclusion criteria

Those neonates whose mothers died and twin neonates were excluded from the study.

### Sampling and sampling procedure

Epi Info 7 software was used to calculate the sample size. The following assumptions were taken into account: 95% confidence level, 80% power of the study, and the case-control ratio of 1:2, percent of exposure among case and control. The percentage of cases exposed to old maternal age (5.4%) and the percentage of controls exposed to old maternal age (15.8%) were taken from a study conducted in Brazil (16). Based on the above assumptions the estimated sample size was 122 cases and 244 controls. After considering the non-response of 10% of the participants, the final sample size used for this study was 134 cases and 268 controls. To recruit the study subject, a consecutive and simple random sampling technique was used for cases and controls, respectively.

### Data collection methods

Data were gathered using a pre-tested, structured, and standard checklist to review information from the medical records and mothers of neonates. The standardized data abstraction was adapted from the tool developed by the London School of Hygiene and Tropical Medicine (LSHTM) and its partners, and is utilized in several multi-country near-miss projects across Francophone Africa. These projects include the World Health Organization (WHO) Multi-Country Survey Project on severe maternal morbidity and the Unmet Obstetric Need (UON) initiatives led by the Institute of Tropical Medicine in Antwerp (16). The checklist includes maternal sociodemographic, reproductive, and obstetric-related variables, maternal medical history during pregnancy-related variables, newborn-related characteristics, and identification criteria for neonatal near-miss conditions. Data were collected by four BSc midwives and supervised by two Masters (MSc) holder midwives.

### Operational definition

#### Neonatal near-miss

NNM was considered when the newborn experienced at least one of the pragmatic or management-based criteria (Table 1).

**Table 1:** Criteria to identify predictors of neonatal near-miss events in Worabe Comprehensive Specialized Hospital, Southern Ethiopia, in 2021.

Criteria	Descriptions
Pragmatic markers criteria	It is the severity of a criterion that determines whether a neonate is classified as a neonatal near-miss. The considered criteria included a birth weight of less than 1,750 g, an Apgar score less than 7 at 5 min, and gestational age of less than 33 weeks (20).
Management severity criteria	It is a criterion based on the management foundation. It includes phototherapy administered within the first 24 h of life, any intubation, nasal CPAP, and parenteral antibiotic therapy. CPR, the use of vasoactive medications, anticonvulsants, surfactants, blood products, steroids for the treatment of refractory hypoglycemia, surgery, the use of antenatal steroids, the use of parenteral nutrition, the identification of congenital malformation in accordance with the international classification of diseases 10th revision (ICD-10) if a near-miss case by another criterion, and admission to the NICU (20).

### Data quality control

Before the actual data collection, the tool underwent a pre-test on 5% of the sample size to evaluate its simplicity, flow, and consistency. During this pre-test phase, the applicability of the instruments was assessed, and feedback from this process was integrated into the final tool to enhance its quality. Before commencing data collection, data collectors and supervisors underwent a 2-day training session on data collection procedures. Throughout the data collection period, continuous follow-up and supervision were conducted by both the supervisor and the principal investigator.

### Data processing and analysis

The data were entered into Epi-Data version 3.1 and exported to Stata version 14 for statistical analysis. Both descriptive and analytical techniques were employed. The study's findings were reported by mean, standard deviation, tables, and figures. Bivariable and multivariable Binary Logistics regression was used to identify predictors of the neonatal near-misses. Explanatory variables with a *p*-value <0.25 during bivariable analysis were entered into multivariable logistic regression. The multicolinearity test was carried out to see the correlation between independent variables using variance inflation factor (VIF) and tolerance test; no variables were observed with tolerance test <0.1 and VIF of >10. The model fitness was checked using the Hosmer–Lemeshow test (*p* = 0.78). Crude and adjusted odds ratios with a 95% confidence interval (CI) were estimated, and variables with a *p*-value <0.05 in the multivariable regression analysis were taken as significant predictors of neonatal near-misses.

## Results

### Sociodemographic characteristics

Altogether 402 mothers with neonates (134 cases and 268 controls) were successfully interviewed, giving a 100% response rate. The mean age of cases was 27.8 years with a standard deviation of 5.2, while the mean age of controls was 28.9 with a standard deviation of 5.3 years. In 76 (56.7%) of the cases, the neonates were girls and for 134 (50%) of the controls, the neonates were girls. Regarding the mother's educational status, 57 (42.5%) of the cases and 102 (38.1) of the controls did not have any formal education. About 84 (62.7%) of the case mothers and 97 (36.2%) of the control neonates’ mothers were rural residents. About one-third or 39 (29.1%) of these case mothers and 78 (29.1%) of the control mother earn more than 3,500 Ethiopian Birr (Table 2).

**Table 2:** Maternal sociodemographic and newborn-related characteristics in Worabe Comprehensive Specialized Hospital, Southern Ethiopia, 2021.

Variables	Category	Cases (*n* = 134)	Controls (*n* = 268)
Sex of the newborn	Male	58 (43.3)	134 (50.0)
	Female	76 (56.7)	134 (50.0)
Maternal educational level	No formal education	57 (42.5)	102 (38.1)
	Primary education	34 (25.4)	78 (29.1)
	Secondary educational	18 (13.4)	45 (16.8)
	Collage and above	25 (18.7)	43 (16.0)
Maternal age categories	15–24	52 (19.4)	29 (21.6)
	25–34	169 (63.1)	88 (65.7)
	≥35	47 (17.5)	17 (12.7)
Maternal place of residence	Rural	84 (62.7)	97 (36.2)
	Urban	50 (37.3)	171 (63.8)
Maternal occupation	Housewife	55 (41.1)	102 (38.1)
	Merchant	46 (34.3)	78 (29.1)
	Government employee	22 (16.4)	62 (23.1)
	Others^a^	11 (8.2)	26 (9.7)
Family income level (ETB)	<1,500	36 (26.9)	123 (45.9)
	1,500–3,500	59 (44.0)	67 (25.0)
	>3,500	39 (29.1)	78 (29.1)

ETB, Ethiopian Birr.

^a^ Student, daily labor.

### Maternal and child health-related factors

Of the neonates’ mothers, 65 (41.0%) of the cases and 163 (68.3%) of the controls had multipara (birth order ≥2). Of the neonates’ mothers, 78 (58.2%) of the cases and 227 (84.7%) of the controls had ANC follow-up visits and about 67 (59.8%) of the cases and 91 (61.9%) of the controls had incomplete (<4) ANC visits. Regarding abortion, a majority of 24 of the neonate mothers of the cases (17.9%) and 32 of the controls (11.9%) had a previous history of abortion. Of the neonates’ mothers, 34 (25.4%) of the cases and 26 (9.7%) of the controls had APH, and 55 (41.0%) of the cases and 48 (17.9%) of the controls had premature rupture of membrane (PROM). Regarding fetal presentation, about 84 (62.7%) of the cases had non-vertex presentation, whereas 73 (27.2%) of the controls had a non-vertex presentation (Table 3).

**Table 3:** Maternal and child health and obstetric factors of mothers in Worabe Comprehensive Specialized Hospital, Southern Ethiopia, 2021.

Variables	Category	Cases (*n* = 134)	Controls (*n* = 273)
Gravidity	Primigravida	47 (35.1)	72 (26.9)
	Multigravida	87 (64.9)	196 (73.1)
Parity	Primiparous	69 (59.0)	105 (31.7)
	Multipara	65 (41.0)	163 (68.3)
ANC	Yes	78 (58.2)	227 (84.7)
	No	56 (41.8)	41 (15.3)
Frequency of ANC follow-up (*n* = 259)	1–3 times	67 (59.8)	91 (61.9)
	4 and above	45 (40.1)	56 (38.1)
History of abortion	Yes	24 (17.9)	32 (11.9)
	No	110 (82.1)	236 (88.1)
Referred from another health facility	Yes	91 (67.9)	125 (46.6)
	No	43 (32.1)	143 (53.4)
Mode of delivery	SVD	63 (47.0)	183 (68.3)
	Instrumental	11 (8.2)	28 (10.4)
	Cesarean delivery	60 (44.8)	57 (21.3)
APH	Yes	34 (25.4)	26 (9.7)
	No	100 (74.6)	242 (90.3)
PROM	Yes	55 (41.0)	48 (17.9)
	No	79 (59.0)	220 (82.1)
Dystocia	Yes	28 (20.9)	47 (17.5)
	No	106 (79.1)	221 (82.5)
History of hypertension during pregnancy	Yes	58 (43.3)	66 (32.7)
	No	76 (56.7)	202 (67.3)
DM	Yes	27 (11.2)	22 (8.2)
	No	241 (88.8)	122 (91.8)
Maternal admission to ICU	Yes	31 (30.0)	61 (22.8)
	No	103 (70.0)	207 (77.2)
Fetal presentation	Non-vertex	84 (62.7)	73 (27.2)
	Vertex	50 (37.3)	195 (72.8)
Neonatal birth trauma	Yes	36 (26.9)	53 (19.8)
	No	88 (73.1)	215 (80.2)

SVD, spontaneous vaginal delivery; DM, diabetes mellitus.

### Clinical characteristics of neonatal near-misses

The selection of cases was based on the CLAP’s definition of a neonatal near-miss. Within this definition, the pragmatic criteria were predominant among the two key criteria. Among the pragmatic criteria, the most common newborn issue was gestational age less than 33 weeks, accounting for 68 cases (50.7%), followed by birth weight less than 1,750 g, with 52 cases (38.8%). In terms of management criteria, the majority of cases involved the use of intravenous antibiotics for up to 7 days and before 28 days of life, totaling 45 cases (33.6%), followed by any intubation in 16 cases (11.9%) (Table 4).

**Table 4:** Clinical characteristics of neonatal near-misses among neonates admitted to Worabe Comprehensive Specialized Hospital, Southern Ethiopia, 2021.

Neonatal near-miss events (*n* = 134)	Frequency (%)
Pragmatic criteria	
Apgar scores below 7	44 (32.8)
Infants with a birth weight below 1,750 g	52 (38.8)
Infants born before 33 weeks of gestation	68 (50.7)
Management criteria	
Cardiopulmonary resuscitation	12 (9.0)
Use of anticonvulsant medications	6 (4.5)
Use of phototherapy within the initial 24-h period	13 (9.7)
Administration of intravenous antibiotics within the first 7 days and prior to 28 days of life.	45 (33.6)
Application of corticosteroids to manage refractory hypoglycemia	2 (1.5)
Utilization of nasal continuous positive airway pressure (NCPAP)	15 (11.2)
Any operative intervention	3 (2.2)
Congenital anomaly or malformation	6 (4.5)
Transfusion of blood components or derivatives	6 (4.5)
Endotracheal intubation	16 (11.9)

### Predictors of neonatal near-misses

The bivariable logistic regression showed that maternal age, place of residence, parity, antenatal follow-up visits, maternal referral, mode of delivery, antepartum hemorrhage, the premature rapture of the membrane, maternal hypertension, dystocia, and fetal presentation of the newborn indicated association at *p*-value <0.25.

In multivariable logistic regression analysis, maternal place of residence, ANC follow-up visits, antepartum hemorrhage, the premature rapture of the membrane, and fetal presentation were identified as significant predictors of neonatal near-misses.

The likelihood of NNM occurrence was twice as high among neonates born to mothers residing in rural areas compared with those born to urban residents (AOR: 2.01; 95% CI: 1.31–5.84). Neonates born to mothers who did not have any ANC follow-up contact were three times more likely to experience neonatal near-misses compared with those born to mothers who did have ANC follow-up contacts (AOR: 2.98; 95% CI: 1.77–5.56). Neonates born to women who experienced APH in the current pregnancy were four times more likely to encounter neonatal near-miss events compared with neonates of mothers who did not experience APH in the current pregnancy (AOR: 2.12; 95% CI: 1.18–4.07).

Neonates born to mothers who experienced PROM in their current pregnancy were 2.55 times more likely to encounter neonatal near-miss conditions compared with neonates of mothers who did not have PROM in their current pregnancy (AOR: 2.55; 95% CI: 1.54–5.67). Neonates who had a non-vertex presentation were three times more likely to have an NNM event as compared with those with vertex presentation (AOR: 3.05; 95% CI: 1.93–5.42) (Table 5).

**Table 5:** Predictors of neonatal near-miss in Worabe Comprehensive Specialized Hospital, Southern Ethiopia, 2021.

Variables	NNM		COR (95% CI)	AOR (95% CI)
	Yes	No		
Age categories (years)				
15–24	52 (19.4)	29 (21.6)	1	1
25–34	169 (63.1)	88 (65.7)	1.07 (0.29–2.39)	0.55 (0.138–2.24)
≥35	47 (17.5)	17 (12.7)	1.54 (0.91–3.09)	1.04 (0.22–4.90)
Residence				
Rural	84 (62.7)	97 (36.2)	2.96 (1.92–4.55)*	2.01 (1.31–5.84)
Urban	50 (37.3)	171 (63.8)	1	1
Parity				
Primiparous	69 (59.0)	105 (31.7)	1.64 (1.11–4.75)	1.23 (0.46–3.62)
Multipara	65 (41.0)	163 (68.3)	1	1
ANC follow-up				
Yes	78 (58.2)	227 (84.7)	1	1
No	56 (41.8)	41 (15.3)	3.97 (214–7.78)**	2.98 (1.77–5.56)*
Maternal referral				
Yes	91 (67.9)	125 (46.6)	2.42 (1.56–3.74)	1.17 (0.69–1.97)
No	43 (32.1)	143 (53.4)	1	1
Mode of delivery				
SVD	63 (47.0)	183 (68.3)	1	1
Instrumental	11 (8.2)	28 (10.4)	1.14 (0.53–2.42)	0.50 (0.99–1.97)
Cesarean delivery	60 (44.8)	57 (21.3)	3.05 (1.97–4.85)	0.69 (0.15–3.19)
APH				
Yes	34 (25.4)	26 (9.7)	3.16 (1.80–5.54)**	2.12 (1.18–4.07)*
No	100 (74.6)	242 (90.3)	1	1
PROM				
Yes	55 (41.0)	48 (17.9)	3.19 (2.00–5.07)**	2.55 (1.54–5.67)**
No	79 (59.0)	220 (82.1)	1	1
Hypertension				
Yes	58 (43.3)	66 (32.7)	2.33 (1.50–3.62)	1.90 (0.76–4.05)
No	76 (56.7)	202 (67.3)	1	1
Dystocia				
Yes	28 (20.9)	47 (17.5)	1.24 (0.73–2.09)	0.87 (0.44–1.71)
No	106 (79.1)	221 (82.5)	1	1
Fetal presentation				
Non-vertex	84 (62.7)	73 (27.2)	4.49 (2.72–7.62)**	3.05 (1.93–5.42)**
Vertex	50 (37.3)	195 (72.8)	1	1

COR, crude odds ratio; SVD, spontaneous vaginal delivery

* *p* < 0.05.

** *p* < 0.001.

## Discussion

Assessing newborn near-misses and predictors can help to prevent neonatal deaths (21, 22). As a result, the purpose of this research was to find out what factors influence neonatal near-misses among neonates admitted to public hospitals in Southern Ethiopia. Maternal place of residence, ANC follow-up visits, antepartum hemorrhage, premature rupture of membrane, and fetal presentation were identified as significant predictors of neonatal near-misses.

The maternal place residence showed a significant association with neonatal near-misses. Our finding is consistent with that of the studies reported in Debretabor (23) and East Wollega (24). The possible reason for the association of place of residence with increased odds of near misses might be the difference in access and quality of healthcare service and health information across residences; this could lead to a delay in accessing necessary emergency, obstetric, and neonatal healthcare. This study infers that improving access to maternal and newborn healthcare services in rural parts will help to decrease the episodes of neonatal near-misses.

Moreover, the odds of neonatal near-misses were more than three times higher among mothers who had not had ANC follow-ups during the current pregnancy. The findings of this study were supported by the studies undertaken in Ambo, Central Ethiopia (25), Gamo Gofa, Southern Ethiopia (26), Tigray region, Northern Ethiopia (27), and Southeast Brazil (28). This might be because during ANC follow-up visits adequate screening and management of high-risk pregnancies and obstetric complications can be done, which consequently, decreases the rates of neonatal near-misses (29). Moreover, pregnant women who had no ANC visits may not receive adequate health information regarding warning signs of pregnancy complications and often only go to a health facility after encountering difficulties during labor. This leads to the infants being more likely to have severe life-threatening conditions and dying during the neonatal period (30). These findings imply that community health extension workers and Woreda Health office administrators should work on the awareness creation about the importance of ANC follow-up visits.

APH also showed a significant association with NNM. The findings were in line with research conducted in Debretabor General Hospital, Northern Ethiopia (23), Southeast Brazil (28), and Harare, Zimbabwe (31). This is because fetal circulation is dependent on the placenta, and antepartum hemorrhage affects fetal blood perfusion, which leads to neonatal morbidity (23).

In this study, premature rupture of membranes was found to be strongly linked to NNM instances. This was in line with studies done in Gamo Gofa, Southern Ethiopia (26), Debretabor General Hospital, Northern Ethiopia (23), and Southeast Brazil (28). This could be attributed to the fact that premature rupture of membranes often triggers preterm labor, which poses risks such as birth asphyxia, chorioamnionitis, neonatal sepsis, pulmonary hypoplasia, and cord prolapse. Various studies have demonstrated a significant increase in the risk of maternal, fetal, and neonatal morbidity and mortality due to obstetric complications associated with premature rupture of membranes (32).

This study indicated that non-vertex fetal presentation during delivery increased the risk of developing neonatal near-misses. This is in line with the findings of a study done in Jimma Zone, Southwest Ethiopia (33) and Gamo Gofa, Ethiopia (26). This may be the case because malpresentation during pregnancy and labor increases the risk of birth hypoxia, birth trauma, and other complications and leads to obstructed and prolonged labor, which can result in neonatal morbidity and mortality (34).

### Strengths and limitations of the study

The paramount significance of this study for public health lies in its identification of potential factors predisposing newborns to life-threatening (near-miss) conditions. This insight is crucial for various stakeholders within the healthcare system to address underlying causes promptly and provide effective remedies. Moreover, the study employed verified and standardized criteria for identifying neonatal near-miss cases, minimizing the risk of misclassification.

However, despite its strengths, the study faces several limitations. Due to its design, controlling for confounding variables proves challenging as cases and controls are not matched with relevant factors. In addition, the use of secondary data posed difficulties in accessing certain vital variables such as maternal wealth index and cultural aspects. This limitation may impede our ability to fully address the short- and long-term consequences of neonatal near-miss events occurring after neonatal discharge.

## Conclusion

In the current study, maternal place of residence, ANC follow-up visits, antepartum hemorrhage, the premature rapture of the membrane, and fetal presentation were identified as significant predictors of neonatal near-miss events. Furthermore, raising awareness about the necessity of full-time ANC attendance, expanding high-quality healthcare facilities to remote areas, and providing individualized obstetric counseling for pregnant women are all important initiatives. High-risk mothers must be identified, screened, and closely monitored, including those who had various complications during previous pregnancies like; having a history of abortion, antepartum hemorrhage, premature rupture of membranes, and non-vertex fetal presentation. Therefore, local health planners and healthcare professionals must work together to improve maternal healthcare services, particularly in terms of early detection of problems and proper therapy.

## Data Availability

The original contributions presented in the study are included in the article/Supplementary Materials, further inquiries can be directed to the corresponding author.
